# Evaluation of porcine reproductive and respiratory syndrome stabilization protocols in 23 French Farrow-to-finish farms located in a high-density swine area

**DOI:** 10.1186/s40813-017-0058-1

**Published:** 2017-05-23

**Authors:** Pauline Berton, Valérie Normand, Guy-Pierre Martineau, Franck Bouchet, Arnaud Lebret, Agnès Waret-Szkuta

**Affiliations:** 1 Porc. Spective, Chene Vert Conseil Veterinary Group, Noyal-Pontivy, France; 2 IHAP, Université de Toulouse, INRA, ENVT, Toulouse, France

**Keywords:** PRRSV, Stabilization, France, Farrow-to-finish, MLV vaccine, Genotype I, Mass vaccination, Herd closure

## Abstract

**Background:**

Porcine reproductive and respiratory syndrome virus (PRRSV) is responsible for reproductive disorders in sows and respiratory problems in pigs, and has a major economic impact. Controlling PRRSV is therefore a priority for the swine industry. Stabilization of a herd, defined as the production of PRRSV-negative pigs at weaning from seropositive sows, is a common method of control, and different protocols have been described in the literature to achieve this stabilization.

**Context and purpose:**

The objective of this study was to evaluate wether the combination of mass vaccination of sows and their piglets with a Genotype I modified live virus (MLV) vaccine, with temporal closure to the introduction of replacement animals and unidirectional pig and human flow can result in the production of PRRSV-negative pigs at weaning. The study took place in French farrow-to-finish farms located in a high-density swine area where the disease concerns over 60% of farms and only closely related strains of genotype I have been reported. Twenty-three 100-to-700 sow farrow-to-finish farms were selected prospectively between 2005 and 2014, regardless of their biosecurity level. Those farms adopted a stabilization protocol characterized by the following standardized measures: vaccination of sows, gilts, and piglets with the Genotype I MLV vaccine PORCILIS®PRRS, temporary herd closure, and strict internal biosecurity measures. Monitoring of herd status was then performed using a combination of 3 diagnostic tools: Real-time polymerase chain reaction (RT-PCR), enzyme-linked immunosorbent assay (ELISA), and Open reading frame (ORF) 5 and ORF7 sequencing. The status of finishing units (either active or inactive, meaning PRRSV-positive or PRRSV-negative, respectively) was not considered in this study.

**Results and conclusions:**

At the end of the monitoring period, considering the results of all the analyses, clinical signs, and epidemiology, 19 farms were considered stable and 1 remained unstable. In 3 farms it was commonly agreed to extend the number of vaccinated batches of piglets, which enabled them to be considered stable at the end of a second round of monitoring. The combination of vaccination of sows and their piglets with a Genotype I MLV vaccine, together with the closure of the farm and a unidirectional pig and human flow, seems to be effective for farrow–to-finish farms even in high-density swine area, even with French PRRSV strains closely related to one another. This research is the first European study examining such a large number of farms, and increased confidence in the results stems from the added value of using the ORF7 and ORF5 sequencing tool.

## Background

Porcine reproductive and respiratory syndrome virus (PRRSV) is classified within the genus *Arterivirus*. Two genotypes are recognized: type I (“European”) and type II (“American”). Within each genotype, many strains exist, grouped into clusters. In France only closely related strains of genotype I are present [1], localized in Brittany where 70% of French pork production is settled, and concern over 60% of farms [2]. In North America, both genotypes are present and can simultaneously co-infect the same farm [3]. The virus, regardless of genotype, is responsible for reproductive disorders of sows and respiratory problems of pigs [4], with dramatic consequences on the animals’ performance. As its economic impact is major [5], controlling PRRSV is a priority in production basins. Eradication of the virus is difficult and requires significant amounts of time and money. Therefore, herd stabilization defined as producing PRRSV-negative piglets from PRRSV-positive sows is a common solution in North America [6]. The most reported method consists of exposing all animals on a farm to a replicating PRRSV, either with an MLV vaccine or by inoculation of a live-resident virus. Exposure is combined with strict internal and external biosecurity measures and herd closure [7, 8]. In Europe, to our knowledge, only a few individual case reports using this method are available [9, 10]. The objective of this study was to evaluate whether a stabilization protocol combining mass vaccination of sows and their piglets with a Genotype I modified-live virus (MLV) vaccine, together with closure of the farm and a unidirectional pig and human flow results in the production of negative pigs at weaning in French farrow-to-finish farms located in a high-density swine area.

## Case presentation

### Herd selection

An association of veterinarians sharing common standards concerning PRRSV diagnosis, control and monitoring selected 23 farrow-to-finish farms located in Brittany purposively and prospectively to be enrolled in a stabilization protocol that was conducted between 2005 and 2014. Bases for inclusion were the willingness of producers to participate in the study and diagnosis of the farm as being unstable. Diagnosis was based on suggestive clinical signs associated with laboratory analyses: when no PRRS MLV vaccine was formerly used on the breeding herd, serology performed on sows confirmed clinical suspicion; when an MLV vaccine was used, RT-PCR and sequencing of a wild strain was performed on blood samples of due-to-wean piglets.

### Study sample

The size of the herds ranged from 100 to 700 sows with a mean of 384 sows (95% CI: 326–442) (Table 1). Study herds followed batch-production practices with farrowing scheduled every 1, 2, 3, 4, 5 and 7 weeks. Farrowing scheduled every 3 weeks with weaning at approximately 28 days of age was the most common (16 of the 23 farms). Most of the producers bought semen (91%, *n* = 21) from serologically monitored boar studs (PRRSV-negative status), and 2 farms practiced on-farm collections. Natural mating was encountered sporadically on 8 farms.

**Table 1:** Characteristics of the farms included in the study

Farm	Year of inclusion	Number of sows	Farrowing schedule (weeks)	Age at weaning	Biosecurity level before inclusion	Mass vaccination of fattening units	Number of vaccinated batches of piglets
1	2012	320	3	28	High	Yes	9
2	2007	290	3	28	Medium	Yes	7
3	2010	320	5	21	Low	Yes	6
4	2007	200	3	28	Medium	No	5
5	2013	240	3	28	Low	Yes	9
6	2013	160	3	28	Low	Yes	8
7	2014	300	2	21	High	Yes	11
8	2014	120	3	28	High	No	8
9	2005	110	3	28	High	No	6
10	2013	500	2	21	High	Yes	6
11	2013	120	3	28	Low	Yes	9
12	2013	420	2	21	High	Yes	13
13	2007	170	3	28	High	Yes	8
14	2007	100	7	28	Medium	Yes	3
15	2008	100	7	28	Medium	Yes	5
16	2010	230	3	28	Medium	Yes	9
17	2013	700	1	21	High	Yes	10
18	2014	240	4	21	Medium	Yes	8
19	2014	250	4	21	High	Partially	10
20	2012	300	2	21	High	Yes	10
21	2007	300	3	28	High	No	10
22	2009	210	3	28	High	Yes	6
23	2013	250	4	21	Medium	Yes	8

### Biosecurity level

Initial biosecurity levels of farms were not a determinant for inclusion in the protocol. They were assessed as high, medium or low by a veterinarian, based on the consistency of housing organization and on observations and discussions with the farmer during visits. Farms were classified as having a high biosecurity level when housing organization implied a separation between sows and post-weaning/fattening facilities and when the farmer respected one-way circulation of people and animals from most-free to contaminated areas (unidirectional flow) with different clothes for different areas. Moreover, in these farms there should be no batch mixing and compliance with all-in/all-out practices. If either of these requirements was deficient, the level of biosecurity of the farm was classified as medium, and if two or more requirements were not satisfied, the level of biosecurity was set to low. For example, a farm in which the producer did not comply with the unidirectional flow of people, used common material for sows and fatteners, wore the same clothes, and where cleaning and disinfection were not systematic between each batch was considered to have a low level of biosecurity.

### Stabilization protocol

Study farms implemented the following protocol:

- Mass vaccination was performed at one time for all breeders at the start (Day 0) of the protocol and 1 month later. Vaccination was then conducted on a 16–20-week interval, depending on the management constraints of the farm.
- Herd closure, meaning no gilt introduction for at least 8 weeks after the implementation of the first mass vaccination, was implemented. At the termination of herd closure, naïve gilts and boars, meaning animals coming from a PRRSV-negative breeding herd, were exclusively introduced.
- Then naïve gilts and boars were vaccinated in quarantine twice at 1-month interval.

In parallel the following 2 measures were also implemented for piglets:

- At the beginning of the protocol (Day 0) the mass vaccination of all growing pigs aged from 21 or 28 days (depending upon age at weaning) to 70 days (before entrance in finishing units) twice at 1 month interval, was performed simultaneously with sows. The mass vaccination of pigs in fattening units (from 70 days of age to slaughter) was not compulsory but depended on the farmer’s objective and on factors that could decrease or increase the chances of success of the protocol, such as the configuration of the premises, global sanitary status of the farm and the level of confidence of the veterinarian regarding compliance with biosecurity measures.
- Additionally batch-to-batch vaccination was conducted at weaning and 3 to 4 weeks later, depending on batch-production practices.

The number of vaccinated batches of piglets generally corresponded to a cycle of production. However, this number could again be higher or lower regarding the farmer’s objectives and factors that could decrease or increase the chances of success of the protocol, such as the configuration of the premises, global sanitary status of the farm, and the level of confidence with respect to biosecurity measures.

All the vaccinations utilized the Genotype I MLV vaccine PORCILIS®PRRS (Lelystad-like, DV strain, MSD Animal Health, Boxmeer, Netherlands), including naïve gilts and boars in quarantine after the termination of herd closure.

Throughout the protocol, strict internal biosecurity measures were implemented, including but not limited to unidirectional pig and human flow, the cleaning and disinfection of facilities where pigs remained or passed through, the prohibition of natural mating and the use of one needle per sow and one needle per 10 pigs. Compliance with the implemented biosecurity measures was assessed based on practices producers pledged to follow and observations made by the veterinarian during farm visits. At least 2 follow-up visits were conducted during the time of the protocol.

The ending of batch-to-batch vaccination of the piglets indicated that the stabilization protocol ended and monitoring could be scheduled.

**Fig. 1:**
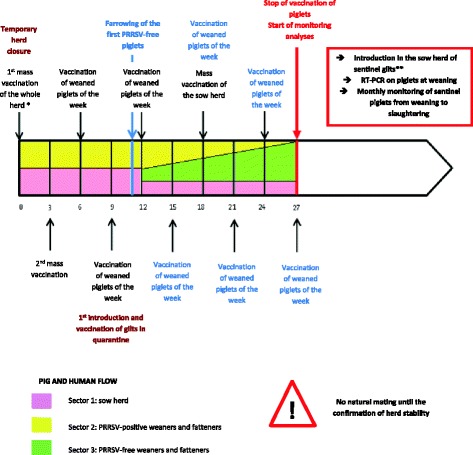
Chronology (weeks) of a stabilization protocol with farrowing scheduled every 3 weeks

An example of a stabilization protocol with the most-encountered French farrowing schedule [11] is presented in Fig. 1.

Eighteen farms agreed to also mass-vaccinate all the animals in the fattening unit present on site at the same time as sows and piglets (Day 0) to maximize the probability of protocol success according to their level of biosecurity (Table 1). In one farm (farm 19), the fattening unit was only partially vaccinated; swine in a barn isolated from the others that had tested negative when the initial status had been checked were not vaccinated.

### Stabilization monitoring

Two-step monitoring was implemented after the termination of batch-to-batch vaccination of piglets [12].

Step 1: Control of virus transmission from the sows to their piglets: RT-PCR (QIamp® Viral RNA Mini Kit, Qiagen, and ADI132-100 - Adiavet PRRSV EU/NA real time 100R) was used to analyse blood samples for 1 to 3 successive batches, depending on farm size. In each batch, a convenience sample of 30 piglets at weaning was used, with each individual belonging to a different litter. Sera were pooled in samples of 3 or 5 (pooling by 3 was used until 2011, then the pooling by 5 method was validated and used for cost concerns).

Step 2: Monitoring of sentinel gilts: non-vaccinated naïve gilts were introduced to the sow herd (gestation and insemination units) at arrival with nose-to-nose contact with sows, at a relation of 1 sentinel gilt per 100 sows. Blood from those gilts was sampled every 2 weeks for 8 weeks and tested serologically (Herdcheck PRRS ×3). If the results were positive, RT-PCR analysis was systematically performed on those sera. Monitoring necessarily began at least 11 weeks after the last mass vaccination of the sow herds, to avoid contamination by the vaccine strain. At the end of the monitoring period, sentinel gilts were vaccinated twice, 4 weeks apart, with PORCILIS®PRRS.

In the case of a positive RT-PCR result, the sequencing of ORF 7 or 5 was implemented to distinguish vaccine from wild strains. ORF7 sequencing was the only available tool in France until 2012. Then, the sequencing of ORF5 was implemented for cost reasons.

During each sampling day, a clinical assessment was performed by observation of animals and discussion with the farmer, looking for suggestive clinical signs of PRRS (low fertility rate or prolificacy, high rate of stillborn piglets, respiratory disorders in fatteners, and high losses, among other signs). In addition, adherence to biosecurity recommendations was assessed during sampling visits.

## Results

Table 2 presents the results for each farm. In farms 1, 2, 3, 6, 8, 9, 11, 12, 15, 17, 19, 20, 21, and 22, all PCR and serological results were negative.

**Table 2:** Laboratory and clinical results

Farm	Due-to-wean piglets (21 or 28 days of age)		Gilts	Biosecurity	Suggestive clinical signs
	PCR positive results	Strain typing (homologous percentage in ORF7 or ORF5)	Positive serological/virological results during follow-up	Adherence to recommendations	Reproductive disorders, respiratory issues, high losses, and/or coinfections
1	No	Not tested^a^	No	Yes	None^c^
2	No	Not tested^a^	No	Yes	None^c^
3	No	Not tested^a^	No	Yes	None^c^
4	No	Not tested^a^	Yes/No	Yes	None^c^
5	Yes	Wild strain (ORF5 96%)	Not tested^b^	No	Yes
6	No	Not tested^a^	No	Yes	None^c^
7	No	Not tested^a^	Yes/No	Yes	None^c^
8	No	Not tested^a^	No	Yes	None^c^
9	No	Not tested^a^	No	Yes	None^c^
10	No	Not tested^a^	Yes/No	Yes	None^c^
11	No	Not tested^a^	No	Yes	None^c^
12	No	Not tested^a^	No	Yes	None^c^
13	Yes	Vaccine strain (ORF7 100%)	No	Yes	None^c^
14	Yes	Wild strain (ORF7 96%)	Not tested^b^	Partially	None^c^
15	No	Not tested^a^	No	Yes	None^c^
16	No	Not tested^a^	Yes/No	Yes	None^c^
17	No	Not tested^a^	No	Yes	None^c^
18	Yes	Wild strain (ORF7 95.09%)	Not tested^b^	Partially	None^c^
19	No	Not tested^a^	No	Yes	None^c^
20	No	Not tested^a^	No	Yes	None^c^
21	No	Not tested^a^	No	Yes	None^c^
22	No	Not tested^a^	No	Yes	None^c^
23	Yes	Wild strain (ORF5 88%)	Not tested^b^	Partially	None^c^

^a^PCR results being negative, no sequencing could be performed

^b^If a wild strain had been identified in piglets at weaning, monitoring of sentinel gilts was cancelled

^c^Clinical assessment was performed at every sampling time

Five farms demonstrated positive RT-PCR results at weaning. In Farm 13, a vaccine strain 100% homologous to the reference vaccine strain was identified after the mapping of ORF 7. In Farms 5, 1, 18, and 23, wild PRRSV strains were identified.

In farms 4, 7, 10, and 16, sentinel gilts were found to be seropositive during monitoring. Samples from these gilts were then tested by RT-PCR, and all results were negative. Therefore, no strain comparison could be performed.

Adherence to biosecurity recommendations was respected on all farms except for farm 5 (no disinfection of barns, no change of clothes between sow and fattener facilities). On farms 14, 18, and 23, recommendations were only partially respected (pig and human flow was not respected properly on all occasions).

No clinical signs suggestive of PRRS disease were noted except in farm 5 where high losses, respiratory disorders, low fertility and prolificacy were encountered.

At the end of the monitoring period, 15 farms (1, 2, 3, 6, 8, 9, 11, 12, 13, 15, 17, 19, 20, 21, and 22) were considered stable.

In farm 5, the lack of respect for both pig and human flow and vaccination schedule led to persistence of the virus during the protocol as well as suggestive clinical signs. Thus, the farm remained unstable despite the efforts taken.

In farms 14, 18 and 23, initial results in piglets were positive and revealed a lack of biosecurity. However, as there were no suggestive clinical signs and as production performances had increased since the beginning of the protocol, it was mutually agreed to extend the number of vaccinated batches of piglets (3, 5, and 5 other vaccinated batches from farms 14, 18 and 23, respectively). This enabled the 3 farms to be considered stable at the end of a second round of monitoring, including another sampling of due-to-wean piglets, and, sentinel gilts.

In farms with sentinel gilts that were seropositive (n°4, 7, 10, 16), all the sampled sera were tested by RT-PCR; however results were negative, so there was no possible identification of the virus. Moreover, production performances of those farms were increasing and no clinical signs were reported. It was determined that in farm 16, the sentinel gilts had been launched less than 10 weeks after the last mass vaccination of the herd. In farms 4 and 7, sentinel gilts had accidentally been in close contact with recently-vaccinated animals. In farm 10, only one serum sample was found to be positive, but the S/P ratio remained unchanged during the whole monitoring period, and no other sentinel gilts became seropositive despite their proximity. In those 4 farms, 10 finishers were blood-sampled, and monthly serological testing from 70 days of age to slaughter, conducted simultaneously, showed no circulation of PRRSV on finishers (in farms 7 and 10, this monitoring was repeated once, given their batch production system with farrowing every 2 weeks, and results were negative the second time as well). Additional ELISA testing was conducted 1 year later in 15 due-to-slaughter pigs, with negative results. Moreover, in farm 7 two batches of 30 due-to-wean piglets were RT-PCR-tested on the occasion of another study, and the results remained negative. The 4 farms were then declared stable.

## Conclusions

Stability was observed in 15 farms for which the monitoring of due-to-wean piglets and sentinel gilts showed the absence of circulating PRRSV. In one farm, the failure of the stabilization protocol was clear, combining identification of a wild strain in piglets and suggestive clinical signs. The lack of respect for biosecurity measures due to discouragement of the farmer could explain this result. On 3 farms, where pig and human flow was not strictly respected, vaccination protocols needed to be extended for another cycle of production. Those 4 cases highlight the importance of farmer motivation when implementing such a protocol and of surveillance by the veterinarian for sufficient respect of biosecurity measures. In the last 4 farms, exposure but not circulation of PRRSV was observed, and diagnostic tools could provide additional information regarding whether a wild or a vaccine strain had prevailed. Extended serological testing of sentinel finishers concomitant with stabilization monitoring and later serological testing of due-to-slaughter pigs, in combination with good production performances and the absence of suggestive clinical signs, enabled us to conclude that those 4 farms were stable.

Vaccination of the fattening units the year of implementation of the stabilization and size of the herd did not seem to be predictive of the success of the stabilization protocol, contrary to adherence to biosecurity measures.

This monitoring and its conclusions face limitation, given that there was no random sampling, the number of sampled animals was arbitrary, and interference by the vaccine strain could complicate diagnoses. As no laboratory test is perfect, both studies in the USA and our own experience lead us to consider that this monitoring along with clinical evaluations, epidemiology and evolution of performances, must be considered in case diagnostic tools are unable to provide clear information.

Three interesting conclusions arise from this field study. First, the measures taken appear relevant as they appear to have enabled the stabilization of the majority of the farms included (*n* = 19). The results of RT-PCR on farms at weaning highlight the importance of implementation of strict biosecurity measures and respect of vaccination schedules. Second, combining 3 diagnostic tools (ELISA, RT-PCR, and sequencing of ORF7/ORF5) appears worthwhile in our context to obtain a clear picture of the stability status of a farm, as it has been previously demonstrated in the USA [12], although their recommendations could not be completely followed [10, 12, 13] due to specific field constraints and costs. Finally, this study reflects the fact it is possible to stabilize a farm independently of its size, localization or herd management system.

Combining vaccination of sows and their piglets with a Genotype I MLV vaccine, along with the closure of the farm and a unidirectional pig and human flow seems to be ultimately efficient in farrow-to-finish farms, even in high-density swine area such as Brittany, all the more that French PRRS strains are closely related to one another. Such a protocol can be a great opportunity for pig production areas, where the disease is endemic, whatever the type of farm. One can easily imagine a regional stabilization plan, the most important component being motivation of both farmers and veterinarians. This research is the first European study to include so many farms, and increasing confidence in the results is provided by the added value of using the ORF7 and ORF5 sequencing tool [10].

## Abbreviations

ELISA: Enzyme-linked immunosorbent assay
MLV: Modified live virus
ORF: Open reading frame
PRRS: Porcine reproductive and respiratory syndrome
RT-PCR: Reverse transcription polymerase chain reaction
